# The Role of Bacterial Symbionts in Triatomines: An Evolutionary Perspective

**DOI:** 10.3390/microorganisms8091438

**Published:** 2020-09-19

**Authors:** Nicolas Salcedo-Porras, Claudia Umaña-Diaz, Ricardo de Oliveira Barbosa Bitencourt, Carl Lowenberger

**Affiliations:** 1 Centre for Cell Biology, Development and Disease, Department of Biological Sciences, Simon Fraser University, Burnaby, BC V5A 1S6, Canada; claudia_silvana_umana_diaz@sfu.ca (C.U.-D.); ricardo_de_oliveira_barbosa_bitencourt@sfu.ca (R.d.O.B.B.); clowenbe@sfu.ca (C.L.); 2 Programa de Pós-graduação em Ciências Veterinárias, Instituto de Veterinária, Universidade Federal Rural do Rio de Janeiro, 23890-000 Seropédica, Brasil

**Keywords:** microbiota, Chagas disease, triatomines, microbiome, symbiosis, *Trypanosoma*, Hemiptera

## Abstract

Insects have established mutualistic symbiotic interactions with microorganisms that are beneficial to both host and symbiont. Many insects have exploited these symbioses to diversify and expand their ecological ranges. In the Hemiptera (i.e., aphids, cicadas, and true bugs), symbioses have established and evolved with obligatory essential microorganisms (primary symbionts) and with facultative beneficial symbionts (secondary symbionts). Primary symbionts are usually intracellular microorganisms found in insects with specialized diets such as obligate hematophagy or phytophagy. Most Heteroptera (true bugs), however, have gastrointestinal (GI) tract extracellular symbionts with functions analogous to primary endosymbionts. The triatomines, are vectors of the human parasite, *Trypanosoma cruzi.* A description of their small GI tract microbiota richness was based on a few culturable microorganisms first described almost a century ago. A growing literature describes more complex interactions between triatomines and bacteria with properties characteristic of both primary and secondary symbionts. In this review, we provide an evolutionary perspective of beneficial symbioses in the Hemiptera, illustrating the context that may drive the evolution of symbioses in triatomines. We highlight the diversity of the triatomine microbiota, bacterial taxa with potential to be beneficial symbionts, the unique characteristics of triatomine-bacteria symbioses, and the interactions among trypanosomes, microbiota, and triatomines.

## 1. Introduction

Insects live in complex environments in which they interact with millions of fungi, bacteria, viruses, and parasites. The interactions among insects and microorganisms can be classified over the symbiotic spectrum from mutually beneficial to parasitic. Insects also can act as biological and mechanical vectors of many microorganisms that can cause disease in humans, other animals, and plants. Insects, however, are not limited to bipartite interactions with single microorganisms as they also harbor and interact with a microbiota comprised of many non-pathogenic and potentially pathogenic bacteria, fungi, viruses, and protozoans (= the holobiont) that can affect the insects’ diet, physiology, immunity, and reproduction. In many cases, beneficial symbionts from the microbiota are essential and have allowed insects to expand and adapt to different environments [1,2,3,4]. The abundance, tissue specificity, taxa represented, and location (including intra-or extracellular) of these symbionts and the general microbiota is variable among insects, and in many cases depict tight coevolutionary histories [5,6,7].

There also are differences in how holometabolous and hemimetabolous insects obtain their microbiota and maintain these microbes from immature to adult stages. Whereas nymphs of hemimetabolous insects may carry their microbiota from early nymphal stages through to the adult stages [8,9], the transformation from larva–pupa–adult in holometabolous insects involves the breakdown of the larval GI tract and the construction of the adult GI tract. During this metamorphosis, there is a high expression of antimicrobial compounds that eliminate most gut bacterial symbionts [8,10,11], and can lead to the establishment of a different microbiome in adults [12]. Adult holometabolous insect may need to reacquire most of their microbiota [11,13,14] or to develop strategies to avoid the elimination of existing microbes during molting [15,16,17].

Historically, the study of these interactions has been focused on microorganisms that could be cultured in laboratories and that relied upon microscopic characteristics for identification. In many cases, the identity of these microorganism was imprecise, but their importance as symbionts was well established [5,18]. The development of sensitive high-throughput molecular techniques (i.e., RNAi, Sanger sequencing, RNA-seq, proteomics, and metabolomics) allows us to describe complex insect microbiota with more precision, to decipher and better understand complex insect–symbiont relationships and the evolution of these symbioses, and to develop new strategies to control insect pests and reduce the transmission of vector-borne pathogens that cause disease. This review will focus on recent advancements in our knowledge of the microbiome and microbiota of triatomines (Hemiptera). These insects have been studied extensively and are considered as models for studies on basic insect physiology [19,20,21], and many triatomines are vectors of the protozoan *Trypanosoma cruzi*, which causes Chagas disease in humans and kills over 15,000 people annually [22].

## 2. Insect–Microbe Symbioses

Insect interactions with microbiota are incredibly diverse. While some insects have specialized organs to harbor single, or few, symbiont species, others have diverse and highly variable microbiota. Many symbioses are established with single, or few, microbial species and may involve the development of dedicated insect cells and organs (i.e., bacteriomes, posterior midgut crypts, and mycangia) to harbor specific species of obligate symbionts [5,6,23,24,25,26,27,28,29,30,31]. In these relationships, there is often genomic complementarity of biochemical pathways required for the survival of both interacting parties [32]. The weevil *Sitophilus zeamais*, for instance, relies on nutrients synthesized by its endosymbiont *Sodalis pierantonius* to survive. The weevils prevent activating a systemic antibacterial response against these symbionts by secreting an antimicrobial peptide (AMP) in the bacteriome that degrades the bacteria-derived peptidoglycan that normally activates the innate immune system [5,24,25,26,27,28]. Termites rely on more complex symbioses with dozens of GI tract protist or bacterial species to digest recalcitrant plant tissues [33,34,35], and many of these microorganisms are termite-specific symbionts with a very strong niche specialization [36,37]. Other insects do not rely on specific essential symbionts and have variable and more flexible associations with their microbiota that may be driven by the local environment [38,39,40,41]. In most insects, the microbiota is important or even essential for aspects of digestion, fecundity, fertility, and immunity [42,43,44,45], as rearing axenic insects may be lethal [46,47]. The acquisition of symbionts that form a suitable and functional microbiome is, therefore, crucial for the success of insects [48].

Beneficial symbionts (commensals or mutualists) may be classified as primary (obligate and essential organisms) or secondary (facultative organisms that improve ecological traits and host fitness). Primary beneficial symbioses are commonly established with a small number of microorganisms that are ubiquitous throughout host populations, whereas secondary symbioses often lack a niche specialization, and various microorganisms can fulfill the secondary role [49,50]. Insects with specialized or nutrient-deficient diets (obligate phytophagy or hematophagy) tend to associate with primary symbionts whereas polyphagous and omnivorous insects have more flexible associations, acquire a variable microbiota from their local environment, and may have secondary symbionts. Many primary symbionts are intracellular (endosymbionts), whereas secondary symbionts tend to be extracellular. The mode of acquisition or transmission of symbionts, be it vertical, horizontal, or a combination of these, may reflect the nature of the insect–symbiont interactions. Primary symbionts, such as those found in weevils and aphids, are commonly transmitted vertically through the female germline [50,51,52,53,54]. Horizontal transmission often occurs with environmentally acquired microbes, some of which have the potential to establish secondary symbioses that do not require specialized organs [50,55,56,57].

Insect development also affects the timing of horizontal acquisition of microbiota. Most insects are nearly axenic (germ-free) upon hatching, excluding vertically transmitted microbes, and acquire their microbiota by coprophagy, cannibalism, trophallaxis, or from consuming their own contaminated eggshells [53,58,59,60,61,62]. Holometabolous insects revert to an almost-axenic state during pupation and some of the microbiota is acquired anew by adults from the environment after emergence [8,14,15,62,63,64,65]. In some species, adults may harbor a very distinct microbiota than the immature stages [10,11,12,66,67,68,69] or acquire a very similar microbiota from the environment or conspecifics [13,70,71]. In contrast, in hemimetabolous insects, microbes that are acquired after hatching can be maintained for a lifetime [8,9]. Lastly, gregarious and social insects, either holo- or hemimetabolous, can obtain microbes repeatedly from their conspecifics, selecting and maintaining a specific microbiota [33,34,35,72,73,74,75,76].

## 3. Evolution of Beneficial Symbioses in the Hemiptera

**Figure 1:**
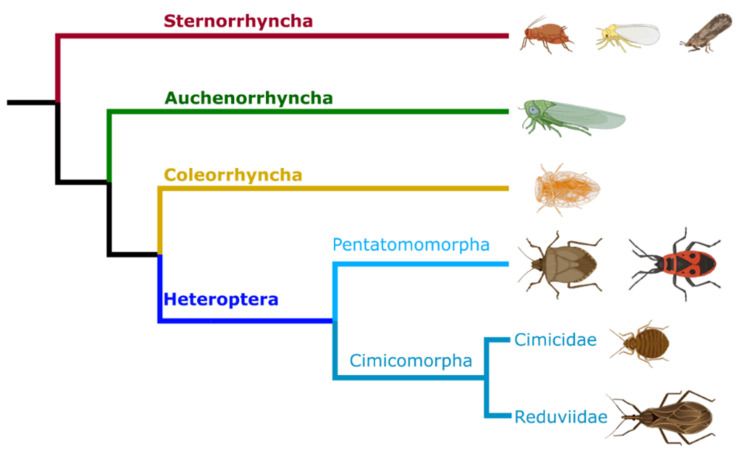
Evolutionary relationships among the four Hemiptera suborders and some selected Heteroptera. Hemipterans are divided taxonomically into four monophyletic suborders: Sternorrhyncha (i.e., aphids, psyllids, and whiteflies), Auchenorrhyncha (i.e., cicadas, spittlebugs, and planthoppers), Coleorrhyncha (i.e., moss bugs), and Heteroptera, (i.e., true tugs). Among the Heteroptera, the infraorders Pentatomomorpha (i.e., stinkbugs and firebugs) and Cimicomorpha (i.e., bed bugs, and triatomines) present symbioses with extracellular GI tract bacteria. Phylogenetic relationships were drawn based on [81,82].

Hemipterans are hemimetabolous insects that evolved ~385 million years ago and represent ~10% of all insects with over 80,000 species [77]. Major adaptations that have contributed to their diversification include mouthparts designed to pierce and suck plant fluids or animal blood. As a result, many hemipterans are pests of crops and some serve as vectors of pathogens for both plants and animals, causing economic losses and human death [22,78,79,80]. Hemipterans are divided taxonomically into four monophyletic suborders: Sternorrhyncha (i.e., aphids, psyllids, and whiteflies), Auchenorrhyncha (i.e., cicadas, spittlebugs, and planthoppers), Coleorrhyncha (i.e., moss bugs), and Heteroptera or true bugs, (i.e., litter bugs, water striders, stinkbugs, bed bugs, and triatomines) (Figure 1) [77]. Each group maintains diverse associations with beneficial symbionts [5].

Many hemipterans feed on plant sap that is an unbalanced source of vitamins, nutrients, and amino acids for insects [83,84,85]. The evolution of associations with intracellular primary symbionts in bacteriomes or extracellular GI tract bacteria addresses these dietary deficiencies and allows the exploitation of new niches [2,5,86]. Most of the Sternorrhyncha, Auchenorrhyncha, and Coleorrhyncha have associations with primary endosymbionts that were established millions of years ago when a few bacterial lineages were selected as primary symbionts [87,88]. These primary symbionts often have reduced genomes and modified metabolic processes that provide for their hosts’ nutritional needs. Over time, however, these ancient associations may lead to the genomic degradation of the symbiont, resulting in the fixation of negative traits (e.g., due to genetic drift) and the loss of the nutritional benefits provided to the host [2,89,90,91,92]. Primary symbioses can therefore change and limit the ecological niche of hosts and potentially may lead to extinction scenarios [3,86]. These disadvantages are believed to have led to the shift and acquisition of new beneficial endosymbionts in some of the Sternorrhyncha and Auchenorrhyncha [2,91,93,94,95], allowing them to remain in their niche feeding on phloem/xylem sap [94,96].

Other associations in the Hemiptera seem to rely exclusively on primary and secondary extracellular symbionts. Extracellular microbes are usually located in the midgut, have wider host ranges, larger genomes with diverse biochemical pathways, and often can survive outside their hosts [3,97]. In the Heteroptera, primary symbioses with extracellular microbes have evolved repeatedly [2,47,53,98,99,100,101,102]. Understanding the driving forces for the evolution of hemipteran–symbiont associations is essential to understand specific triatomine–symbiont associations and interactions.

### 3.1. Sternorrhyncha and Auchenorrhyncha Symbioses

Animals cannot produce up to 10 essential amino acids, which must be acquired from their diet or beneficial symbionts [32,103]. Auchenorrhynchans and Sternorrhynchans feed on amino acid- and nitrogen-deficient plant sap [84]. To supplement these poor nutritional resources, these insects harbor primary endosymbionts that provide the required nutrients [2,32,104,105,106]. In turn, the endosymbionts acquire amino acids from the host that they cannot synthesize [32,107]. This type of association, known as genomic complementarity, requires special co-adaptation strategies to guarantee the inheritance of the symbiont. Almost all primary symbionts in the Sternorrhyncha and Auchenorrhyncha are inherited vertically via transovarial transmission, reside in bacteriomes [5,108], and cannot establish as free-living organisms [2,32]. To avoid the detection and elimination of their symbionts, some aphids have reduced their repertoire of immune receptors and express specific AMPs around bacteriomes to prevent the escape and elimination of the symbiont [86,109,110]. Some holometabolous insect–endosymbiont systems show similar adaptations to tolerate and regulate microbial symbionts [6,111,112].

Symbiotic relationships in the Sternorrhyncha and Auchenorrhyncha represent some of the oldest associations known in insects. In the Sternorrhyncha, most primary symbionts are Gammaproteobacteria whose acquisition can be traced to the evolution of subfamilies in the Precambrian era. *Carsonella* sp. is present in most psyllids and whiteflies and is thought to have been acquired by a common ancestor ~200 mya [88,113]. Aphids are believed to have acquired *Buchnera sp*. as a primary symbiont during the same period [87]. In the rest of the Sternorryncha (i.e., mealybugs and scale insects), other primary symbionts were acquired more recently and might have replaced older symbionts [2,114,115,116,117]. *Sulcia muelleri* (Bacteroidetes) is present in most species in the suborder Auchenorrhyncha and the association can be traced back ~270 mya [77]. While these primary symbionts provide most essential amino acids and nutrients, the recent and variable acquisition of other symbionts provides the remaining nutritional requirements [2,3,28,86,118,119]. These newer primary symbionts comprise diverse taxa and include Gammaproteobacteria, Bacteriodetes, Betaproteobacteria, and fungi [2,114,115,116,117,120,121].

Evidence of these ancient primary symbioses is seen in phylogenetic studies as patterns of co-speciation of insects and their symbionts. Even within a species, maternal lines (matrilines) can be traced to individual bacterial strains [122,123]. These strong and ancient coevolutionary adaptations were originally advantageous for the diversification of the Sternorrhyncha and Auchenorrhyncha [86,119], but over time may create overspecialization and extinction scenarios [2,86,124]. For instance, symbiont vertical inheritance, low genetic diversity, and small bacterial population size favor genetic drift and the fixation of deleterious mutations. These mutations shift the genomic nucleotide composition, generating unstable proteins with narrow temperature ranges that are metabolically costly to stabilize [125,126,127]. Furthermore, endosymbionts show trends towards strong genome size reduction with irreversible gene losses, including some of the nutrient synthesis genes essential for the host [28,124,128,129].

These co-adaptations can result in restricted ecological niches and stimulate the loss and acquisition of new symbionts [115,118,130,131]. If an insect could acquire symbiont-supplemented nutrients from a new source (i.e., diet or new symbiotic associations) then the benefits of maintaining the original symbiont might disappear [2,115,118,130,131]. The loss of symbionts can be exacerbated by “selfish” endosymbionts. Since symbiont fitness is directly related to maternal line success, symbionts benefit from female sex ratio biases, and by increasing their numbers within bacteriomes [86,132]. Some hosts have strategies to tolerate these “selfish” symbionts (i.e., immune pathway reductions or modifications) or to control their reproduction and to prevent their escape from the bacteriome (i.e., local AMP production) [6,133]. If mechanisms to control selfish symbionts fail, and the symbiont is no longer beneficial, then novel symbiont acquisition would be favored. In fact, the more recent acquisition and shift of endosymbionts is common in most of the Sternorrhyncha and Auchenorrhyncha [28]. These new symbionts have genomic complementarity with the previously established symbionts and the insect hosts, restoring and stabilizing the new insect–symbiont-1 (original)–symbiont-2 (new) system [134]. However, these multipartite systems are also subject to degeneration over time [108,114,135].

### 3.2. Heteroptera Symbioses

The Heteroptera is the most diverse hemipteran suborder in terms of species numbers, habitats, and diets; it contains seven infraorders, and most families lack primary symbionts [136]. The ancestral Heteroptera transitioned from a herbivorous diet to predation in most infraorder species and later, to herbivory in a few species of Cimicomorpha and in most species of Pentatomomorpha [136,137,138]. Two infraorders contain most of the Heteroptera (66%) and are exclusively phytophagous, feeding on plant reproductive organs, seeds, and multiple plant vascular tissues that are richer in nutrients and amino acids than sap [139,140]. The five basal Heteroptera infraorders have a predatory or omnivorous lifestyle. Among these, some species have adapted to specialized diets such as vertebrate blood (e.g., kissing bugs (*Rhodnius* sp.) and bed bugs (*Cimex* sp.)), that is richer in dietary nutrients than plant sap [78,141,142], but still lacks some nutrients that are required by the insect and that are supplied by symbionts.

The absence of primary intracellular symbionts in the Heteroptera is likely due to their richer diets. Nonetheless, extracellular primary symbionts are present in the midgut of many Heteroptera, and their removal results in slow or incomplete development, altered foraging behavior, reduced fecundity, and increased mortality [2,46,47,53,59,60,100,143,144,145,146,147,148,149,150,151], suggesting that the symbionts provide significant and essential benefits to their host. Despite their importance, the transmission of primary symbionts in the Heteroptera rarely relies on bacteriomes or transovarial transmission. Instead, hosts have evolved other effective modes of transmission including fecal smearing, coprophagy, social transmission, environmental acquisition, and carnivory [2,97,100,144,146,151]. Other strategies are more complex; some species enclose the symbionts in jelly [152] or capsules that are deposited on, or around, the eggs and later are ingested by first instar nymphs upon eclosion [53,59,60]. These strategies are successful alternatives to the transovarial transmission of beneficial symbionts.

In contrast to primary symbionts of the Sternorrhyncha, Auchenorrhyncha, and Coleorrhyncha, the primary symbionts of most heteropterans can exist as extracellular or even as free-living organisms, have larger genomes, and are not subjected to genomic decay due to genetic drift. Genomic-degeneration hallmarks and patterns of codiversification have only been seen in a few symbionts of the Heteroptera that use jelly or capsules for symbiont transmission, or in some hosts, such as phytophagous pentatomids that have specialized crypts or cecae and accessory organs to select and transmit the symbionts [46,53,59,60,97,142,152,153,154,155,156,157,158]. The degree of genomic decay in these symbionts is less than in primary endosymbionts [59,142,153,155].

Most of these diverse adaptations have evolved independently multiple times in the infraorders Cimicomorpha and Pentatomomorpha [157,159]. The latter group contains agricultural pests and has conspicuous adaptations (e.g., cecae) to harbor primary symbionts in their midguts. However, these adaptations are not shared with other Heteroptera; the family Arabidoidea, which is basal in the Pentatomomorpha, lacks specialized midguts and any known symbionts [100] suggesting that these are derived convergent adaptations in this infraorder [100,151]. Species outside the Pentatomomorpha are largely predators or omnivores with diversified diets and their GI tract microbiota might be less relevant to their development and fitness [160]. The only other heteropteran taxon with evidence of symbiotic associations is the Cimicomorpha (sister clade to Pentatomomorpha) with specialized obligate hematophagous diets. These include the kissing bugs (Reduviidae: Triatominae) that harbor extracellular midgut primary symbionts and the bed bugs (Cimicidae) that have primary endosymbionts [161], but neither of these two groups has morphological adaptations in the GI tract.

The diversity in the Heteroptera provides a spectrum of evolutionary adaptations, including multiple and different beneficial symbiotic associations. The degree of association seems to be related to the level of dietary specialization. Some associations are conspicuous and easy to detect (i.e., midgut crypts, egg capsules, egg jelly, sap-feeding, hematophagy), while others can be inferred only from the effect of artificial dysbiosis. The principal role assigned to these symbionts is nutritional supplementation [152]. Most studies looking at the role of individual symbiont species use broad-spectrum antibiotics to eliminate the major symbiont or symbionts and then evaluate host fitness factors. These antibiotics likely have detrimental effects on almost any microbial symbiont, and therefore the precise role and contribution of each microbial symbiont in the Heteroptera is largely unknown [56,157,162]. Other proposed roles of these symbionts include protection against parasites [163], immune system homeostasis [164], insecticide degradation [165], B vitamin provisioning [166], plant toxin detoxification [153], and dietary niche adaptation [167].

## 4. Triatomine Symbioses

The basal Heteroptera, in particular the infraorder Cimicomorpha, present a striking contrast with the feeding behavior of other hemipterans; many species prey on animals and are polyphagous [141,168]. Although the Cimicomorpha is a diverse group with conspicuous names such as pirate bugs, thread-legged bugs, lace bugs, damsel bugs, web lovers, and bat bugs, most research has focused on the bed bugs (Cimicidae) and kissing bugs (Reduviidae: Triatominae) likely due to their specialized obligate hematophagous diet, their nuisance to humans, and the potential of kissing bugs to transmit parasites to humans and animals [78].

Reduviids are considered to be one of the most diverse and largest predatory groups of animals (>6800 species) [169,170] divided among 25 subfamilies. All known species are polyphagous predators of other animals, except for the subfamily Triatominae [78]. Members of the Triatominae, along with members of the family Cimicidae, are obligate vertebrate-blood feeders and both groups rely upon obligate symbionts to supply nutrients that are scarce or absent in vertebrate blood [5,57,78].

In the Triatominae, GI tract bacteria, including beneficial symbionts, have been known for over 90 years [171]. These insects lack bacteriomes or midgut crypts to harbor bacteria and the extracellular symbionts reside in the lumen of the midgut and hindgut [47,57,171]. Classical studies based on culture dependent methods, revealed a small richness in their intestinal microbiota. Some of these bacteria (e.g., *Rhodococcus rhodnii*, *Nocardia* sp., and *Corynebacterium* sp.) are primary symbionts as they are required for the development and survival of the insects [47,172,173,174,175]. Recent studies using culture independent methods, however, have revealed new interactions with a more complex and diverse microbiota in triatomines (Section 4.2). We will review both classical and recent findings in the next sections.

### 4.1. Classical Studies with Rhodococcus rhodnii

The apparent absence of GI tract microbes in triatomines was refuted by Duncan in 1926 [171] and validated by Brecher and Wigglesworth in 1936 and 1944 [47,57]. They characterized an extracellular bacterium found in the lumen of the midgut and hindgut of *R. prolixus*, *Triatoma rubrofasciata*, *Triatoma infestans*, and *Triatoma flavida*, and suggested it was involved in providing B vitamin supplementation to their hosts [57,176]. This actinobacterium was described initially as *Actinomyces rhodnii* and today as *Rhodoccocus rhodnii* [177].

*Rhodococcus rhodnii* is acquired by triatomines through coprophagy and the primary symbiotic role of this bacterium has been proven repeatedly. Aposymbiotic triatomines (sterile raised and germ-free insects that lack *R*. *rhodnii*) have extended molting times, increased mortality, and abnormal development of limbs and organs [47,98,102,178,179,180]. Aposymbiotic insects develop normally until the 3^rd^ or 4^th^ instar, but most fail to molt to 5^th^ instar, and almost none reach the adult stage [47,98,178,179,180]. *Rhodnius prolixus* that feed exclusively on blood from rabbits that have been immunized against *R. rhodnii* have developmental issues similar to aposymbiotic insects [98]. Aposymbiotic insects that are supplied with *R. rhodnii* revert to the normal condition [47,102,176,181,182] confirming the role of this bacterium as a primary symbiont. While the prevalence of this bacterium is high in lab-reared and wild *R. prolixus*, suggesting it acts as a primary symbiont in this species, other triatomines have a low or zero prevalence of *R. rhodnii* [172,181,183]. *Triatoma infestans*, for example, has a low prevalence of *R. rhodnii* [47,172,176,182] but other bacteria, such as *Corynebacter* sp. and *Nocardia* sp., may act as primary symbionts in *Triatoma* sp. [173,174,176]. Other *Nocardia* sp. and *Rhodococcus* sp. that are not native to triatomines can rescue the aposymbiotic symptoms in *R. prolixus* which suggests that the benefit provided by *R. rhodnii* is shared with other bacteria and that these beneficial roles have not been strongly selected as is seen in other primary symbionts of the Hemiptera [180,184].

Most classic studies on symbionts in the Hemiptera describe bacteria that can be cultured in the laboratory. While the taxonomic identification of some symbionts was questionable, the studies clearly showed a beneficial role of *R. rhodnii*, the deleterious effects of aposymbiosis, and an interspecific variation of the microbiota in triatomines [47,172,176,180,181,183,184,185,186]. Although many culturable and non-culturable microbes inhabit the GI tract of triatomines, only *R. rhodnii* and *Corynebacterium* sp. have been shown to act as true primary symbionts, as they are highly prevalent in their host species, can be transmitted to conspecifics by coprophagy, and they can rescue the aposymbiotic derived symptoms. Current molecular approaches using 16S sequencing of bacterial components of the microbiota of several hemipterans will expand our knowledge of which species are highly conserved and essential as primary symbionts. 

The principal beneficial role proposed for these primary symbionts is the supplementation of B vitamins [57] but confirming this role and identifying the range of nutrients have been difficult. The limitation of triatomine–symbiont models has restricted this research to the *R. rhodnii*-*R prolixus* system. Two experimental approaches have been used: The first approach supplements the diet of aposymbiotic insects with B vitamins and the second one uses auxotrophic mutant bacterial strains (an organism that cannot synthesize a particular compound and that can only grow if the compound is provided externally) to determine if these nutrients are the essential substance provided by the symbiont. The pioneering experiments using the first approach suffered from methodological difficulties, low sample numbers, and conflicting results due to their disparate experimental approaches [102,178,187,188]. The creation of an efficient artificial blood-feeding system has allowed the systematic testing of vitamin supplementation in aposymbiotic *R. prolixus,* resolving some of these issues [178,189]. A combination of the vitamins B1, B2, B3, B5, B6, and B9 restored molting in 4th instar insects. B5-supplementation alone accounted for ~80% of the rescue [178], but very few insects reached the adult stages, suggesting that other factors are provided by the symbionts [178]. In support of these results, recent data from the genome of *R. rhodnii* identified genes required for the *de novo* biosynthesis of these vitamins as well as B7 and B12 [190] and also corroborated earlier biochemical studies on *R. rhodnii* metabolism [178,187,188].

These conclusions were challenged and the difficulties in concluding that B vitamins are the essential nutrient provided by *R. rhodnii* were highlighted [191]. Aposymbiotic insects received vitamin supplementation along with the bloodmeal and not at the time of maximal bacterial population growth in the anterior midgut (~24–48 hours post-feeding [192]). Individual and full factorial supplementation of B vitamins was not done, and the rescue from the aposymbiotic symptoms by vitamin supplementation did not indicate that these are produced by the bacteria [191]. Hill and collaborators [191] opted for a more controllable approach to test the B vitamin supplementation hypothesis by creating *R. rhodnii* auxotrophic mutants. They concluded that vitamins are not the essential factor since insects infected with auxotrophic strains for B1, B2, B3, B6, B7, or PABA developed normally. They proposed instead that symbionts can metabolize vitamin precursors or other substances that aid in the general nutritional requirements for triatomine development. However, the auxotrophic strains themselves were created with imprecise mutagenic approaches; many had leaky phenotypes, lost their auxotrophic phenotype, and were not tested for their ability/inability to produce B vitamins.

The source of vertebrate blood may affect the normal development of the insect, possibly mediated through the symbionts. Aposymbiotic triatomines can develop normally when they are fed on mouse blood [193], but there are negative effects when the blood comes from Guinea pigs, rabbits, sheep, or humans [98,102,178,182,188,193]. The different nutritional profiles of vertebrate blood might explain the variability in earlier studies that supplemented blood with B vitamins. Indeed, the development of triatomines with normal GI tract microbes also varies with the blood source supplied [194,195,196,197,198,199,200,201,202]. The blood source, therefore, can offer different nutritional profiles, contain toxic substances to the insect, and may affect symbionts, especially midgut and hindgut primary symbiont populations, influencing the outcome of experiments. 

Despite these apparently contradictory experimental results, primary symbionts from other hematophagous arthropods do provide B vitamins to their host. The development and reproduction of ticks, lice, Tse-Tse flies, and bedbugs all rely on primary endosymbionts in bacteriomes or mycetomes that supply B vitamins and other nutrients [203,204,205,206,207,208,209,210,211,212,213]. Furthermore, all these primary symbionts have enzymes and genes involved in the de novo synthesis of B vitamins, as does *R. rhodnii* [206,214,215]. The convergent evolution of B vitamin supplementation by primary symbionts is a clear indication of the importance of these nutrients in obligate hematophagous insects [206,214], yet triatomines may be the only obligate hematophagous arthropods that do not rely on primary endosymbionts to supply B vitamins or other essential nutrients.

Perhaps the best example of confirmed B vitamin supplementation by mutualistic symbionts in obligate hematophagous insects occurs in Tse-Tse flies, and their primary endosymbiont *Wigglesworthia* sp. [216,217,218,219]. The bacteriomes of Tse-Tse flies overexpress transcripts for B vitamin synthesis and transport. Proteomic and metabolomic studies indicate that aposymbiotic flies lack B vitamins compared with Tse-Tse flies that harbor *Wigglesworthia* sp. [204,220]. These studies also showed that B vitamins can be interconverted to other B vitamins, which directly and indirectly affects the correct function of a plethora of metabolic pathways [204,220]. These complex and interconnected metabolic networks might help explain the variability seen in triatomine experiments and why it has been difficult to find a specific essential supplemented nutrient in the *R. rhodnii*–*R. prolixus* symbiosis model.

Other parasitic microorganisms such as trypanosomes might affect the availability of B vitamins supplied by bacteria. When *T. infestans* is infected with *B. triatomae* and *R. prolixus* with *T. rangeli* (these microorganisms are only pathogenic to their respective hosts) [221], they experience symptoms similar to aposymbiotic insects. These parasites may reduce the population of midgut symbionts or directly consume the B vitamins, as the symptoms can be ameliorated by supplementing the diet with B vitamins [43,222]. The media in which *T. rangeli* is cultured inhibits the growth of *R. rhodnii* [223], and *T. infestans* infected with *B. triatomae* has a reduced population of midgut bacteria [222,224].

Despite the growing literature, the mechanisms underlying the essential role of symbionts in triatomines and the importance of symbiont-derived B vitamins are still unclear. Improvements in the auxotrophic symbiont mutant approach [191] may now use information in the draft genome of *R. rhodnii* to target B vitamin synthesis with genetic engineering tools [190,191,225]. Similarly, the use of proteomics, metabolomics, and transcriptomics to compare aposymbiotic insects with healthy symbiont bearing insects, as well as studying blood-feeding effects on symbiont population levels, metabolism, and gene expression, will help clarify in much greater detail, the role of symbionts in triatomines [204]. The growing investigation of symbionts in triatomines other than *R. prolixus* will allow for more extensive comparative genomics studies to detect conserved genes and pathways involved in the mutualistic molecular interactions between triatomines and their symbionts [3,28,226].

### 4.2. Microbiome Studies

Beneficial symbioses that involve more than two organisms have received increased attention in recent years, as more data on the beneficial or even essential role of the microbiota in insects emerges. In termites, the GI tract microbes are, as a community, essential to their host, as individual symbionts cannot provide all the required services by themselves [33,35]. The elimination of the microbiota in most insects that do not have primary or secondary symbioses may be detrimental to their host’s fitness but usually is not lethal, suggesting that the normal physiological, developmental, and reproductive processes do not require, but definitely benefit from, microbial symbionts [42,43,44,45].

Classical microbiota studies have dissected tissues from insects, plated microbes on various culture media, and used standard microbiology procedures to identify the organisms that grew; probably identifying only a minute fraction of the total microorganisms present [227,228].

The era of molecular biology, next generation sequencing (NGS), and bioinformatics has allowed us to identify most microorganisms, and their genes (the microbiome) in insects by sequencing highly conserved genomic regions (barcoding) [229]. This approach allows us to measure the microbiota composition quantitatively, usually to the genus level, and to describe its composition in terms of richness (i.e., alpha diversity) and variability (i.e., beta diversity). Although barcodes exist for all taxa, most microbiome studies sequence the 16S ribosomal RNA (16S rRNA) from bacteria. This gene contains multiple variable regions that are useful to detect and identify bacteria and discriminate among closely related taxa [230,231].

In triatomines, the first study to use 16s rRNA, in 1997, identified the intracellular bacterium *Arsenophonus triatominarum* in *T. infestans* [232]. More recently, high-throughput 16S rRNA sequencing has been used to assess complete bacterial microbiomes [9,74,233,234,235,236,237,238,239,240,241,242,243,244,245]. Most studies have focussed on GI tract microbiomes as *T. cruzi* and beneficial symbionts co-reside and develop in its lumen. These studies have described the triatomine microbiome composition [9,74,233,234,235,236,237,239,240,241,242,243,245] and differences associated with developmental stage [9,234,235,236], geographical location and ecological habitat [9,234,237], blood feeding source [235], specific segments of the GI tract [236], the presence of trypanosomes [9,234,235,237,241,245], and differences found among triatomine species [9,240,241]. A direct comparison of these studies is difficult as there is great variation in the source of the tissues used to collect the microbiota (e.g., whole GI tract, feces, abdomen, whole body), the different rRNA sequence markers, the developmental stages used, the vertebrate blood source used to feed the insects, and the times that tissues were collected. Nonetheless, some generalities can be drawn from these studies.

**Figure 2:**
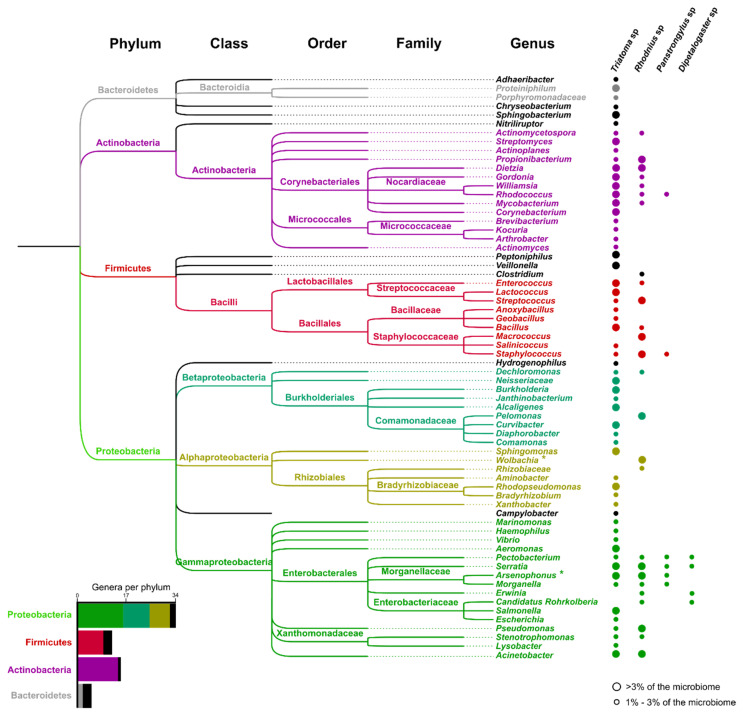
The predominant bacteria from the GI tract of triatomines. Bacteria that represent over 1% of microbiomes from triatomine GI tracts belong to the phylum Bacteroidetes (5 genera in the clade in grey), Actinobacteria (14 genera in the clade in purple), Firmicutes (12 genera in the clade in red), and Proteobacteria (33 genera in the clade in green). Bacteria classes with only 1 representative genus are colored in black. The presence of these bacteria in different triatomines is shown as circles on the right side. Dominant bacteria (1%–3% of a microbiome) are represented with small circles and highly dominant bacteria (>3% of a microbiome) are represented with larger circles. The family Nocardiaceae and the order Enterobacteriales have many highly abundant bacteria. Intracellular bacteria are marked with a *. Data to build this figure were taken from [9,74,233,234,235,236,237,238,239,240,241,242,243]. The phylogenetic tree depicting the evolutionary relationships among bacteria was modified after retrieval from the NCBI taxonomy tool.

The triatomine microbiomes have a microbiota diversity two orders of magnitude greater than previous estimates using culture-based methods (Figure 2). However, these microbiomes have relatively low alpha and high beta diversities compared with other insects [9,74,233,234,235,236,237,239,240,241,242,245]. Most triatomines have fewer than 100 bacterial genera or operational taxonomic units (OTUs), with 4–40 dominant OTUs (>1% of the total reads) comprising most of the microbiome [9,74,233,234,235,236,237,239,240,241,242,245]. In terms of abundance, the microbiome of triatomines is principally composed of the Phylum Actinobacteria (which includes the beneficial Corynebacteriales *Rhodococcus*, *Nocardia*, and *Corynebacterium*) or Proteobacteria (20%–50%) followed by Firmicutes (20%), Bacteroidetes (5%–7%,), and other taxa [9,233,234,235,236,240,242,245] (Figure 2). 

The microbiome composition is very variable among triatomines species and no single bacterial genus is present in all triatomine species, which supports the purported facultative symbiotic role of individual bacterial species. However, some specificity occurs as many genera of Corynebacteriales (principally in the family Nocardiaceae) [9,233,234,235,236,240,242] and Enterobacteriales [9,233,234,237,242] are highly dominant (>3% of the microbiome) and are present across multiple triatomine species, suggesting that these groups have adaptations to withstand the harsh environment of the triatomine midgut and hindgut, or that the host has evolved mechanisms to ensure their survival [239]. Within the Nocardiaceae, the genera *Dietzia* and *Mycobacterium* are present in many populations of *Rhodnius* sp. and *Triatoma* sp. [9,74,233,234,242] and might fulfill beneficial roles more widely than *Rhodococcus* sp. and *Corynebacterium sp*.

In *Rhodnius* sp., Firmicutes are present in a lower proportion than are Actinobacteria, which in some populations is the dominant Phylum [233,240]. In some species, the intracellular bacteria *Wolbachia* sp. (only reported from *Rhodnius* sp.) [233,240] or *Arsenophonus* sp. [9,74,241,242] represent >50% of the total microbiome [243]. Nonetheless, the dominance of Proteobacteria/Actinobacteria in the GI tract is still maintained despite the overrepresentation of *Wolbachia* sp. and *Arsenophonus* sp.

Each triatomine genus and species has a microbiome composition that is distinct from other triatomines [9,235,239,240,241,242,245,246]. In general, members of the genus *Triatoma*, have richer microbiomes than the genus *Rhodnius*, with many bacterial OTUs being highly dominant (>3% of the microbiome). In *Triatoma* sp., 26 genera of bacteria have been reported as highly dominant. Most of these belong to the class Actinobacteria (principally Corynebacteriales), followed by Bacilli (principally Bacillales), Gammaproteobacteria (principally Enterobacteriales), Betaproteobacteria, and Alpha Proteobacteria and Bacteroidia. In contrast, *Rhodnius* spp. have 9 dominant genera in the Actinobacteria, Bacilli, and Gammaproteobacteria.

The triatomine microbiota also changes in both alpha and beta diversity throughout host development stages [9,235,236]. In *R. prolixus*, a diverse microbiome, including bacteria from the surrounding environment, is acquired by coprophagy in first instar nymphs and even the most abundant bacterial genera show different abundance patterns throughout development. *Enterococcus* sp. (Firmicutes), is initially present in small proportions in first instars but increases its numbers during ontogeny, becoming the principal bacterium in adult *R. prolixus*. *Arsenophonus* sp. (Proteobacteria), peaks in abundance in third instars; and *Acinetobacter* sp. has moderate numbers in early instars, but these progressively decrease, reaching their lowest levels in adults. *Rhodococcus* sp. and other Nocardiaceae, are present in all developmental stages but in low proportions [9]. Similar changes in microbiome occur throughout developmental stages of other triatomines [236]. Male and female adult triatomines usually have an overlapping microbiota, and in some species, females have a slightly richer and more diverse microbiota than males [9,235,236,245].

Different regions of the GI tract (anterior midgut, posterior midgut, and hindgut) harbor distinct bacterial microbiomes [236]. In the anterior and posterior midgut of *Rhodnius* sp. and *Triatoma* sp., bacteria of the family Nocardiaceae are dominant. These bacteria increase rapidly in number in the anterior midgut when the insect takes a blood meal, which coincides with a downregulation of host immune factors [247,248,249,250]. These immune factors are later restored and are believed to regulate or modulate uncontrolled bacterial growth. In the anterior midgut, the Nocardiaceae population drops as a consequence of immune and digestive factors [249,250]. These bacteria are believed to be digested, providing other essential nutrients to the host, supporting their putative role as nutritional symbionts [192,247,251].

### 4.3. Effects of Trypanosomes on the Microbiota

The interactions among bacteria, triatomines, and trypanosomes or other parasites or pathogens have been studied extensively. Antagonistic interactions between trypanosomes and symbiotic bacteria have been demonstrated repeatedly. For instance, *T*. *cruzi* alters the triatomine microbiota richness and beta diversity [9,234,235,237,241], and the growth media in which *T. rangeli* is cultured inhibits bacterial growth [223].

The antimicrobial effects of the parasites seem to be related to their pathogenicity and level of virulence. In *R. prolixus* infected with *T*. *rangeli* or *T*. *cruzi.* and in *Triatoma* sp. infected with *B*. *triatomae*, the number of bacteria in the anterior midgut is significantly reduced [224], but not in insects infected with non-pathogenic microorganisms [224]. These results suggest an indirect competition among parasites and bacteria or a direct targeting of the symbionts by the parasites [224].

The coevolution of sympatric trypanosomes and triatomines (species that evolved in the same geographic area) influences the ability of the parasites to infect the host, determines the triatomine immune responses, and the effects on the microbiota. Sympatric trypanosomes that establish successful infections trigger different immune responses than non-sympatric parasites that usually are not infective [252,253,254]. *Rhodnius* sp. are commonly found infected with *T*. *cruzi* from the Discrete Typing Unit (DTU)-I strain and *Triatoma* sp. by *T*. *cruzi* DTU-II, -V, and –VI strains. When *R. prolixus* is fed with *T*. *cruzi* Dm28c (DTU-I) there is an increased expression of some AMPs and significantly reduced numbers of symbiotic bacteria [247,255]. These effects are less noticeable in infections with the Y-strain (DTU-II) which fails to develop in these insects [247,255]. Despite changes in the bacterial population levels, the bacterial diversity in lab-reared triatomines does not change in insects infected with sympatric *T*. *cruzi* strains [241]. There are, however, changes in bacterial diversity when the insects are fed with non-sympatric trypanosomes [241]. Although non-sympatric *T*. *cruzi* also triggers immune responses, these changes seem to affect different bacterial species [241,255]. These results support the idea that sympatric *T*. *cruzi* strains manipulate the triatomine immune responses, altering the microbiome to help the parasite establish an infection. The change in the microbiota caused by non-sympatric *T*. *cruzi* strains may be a collateral result of the immune responses against the trypanosome [241].

Feral insects naturally infected with *T*. *cruzi* have a greater microbiota richness and beta diversity than uninfected feral insects or infected or uninfected laboratory-reared insects [9,234,235,237,241,245] (especially with *Actinomycetes*), and insects infected with different DTUs have a significantly different microbiota composition [245], indicating that the microbiome may also be affected by sympatric trypanosomes [9,233,235,241,245]. The reduced bacterial diversity in laboratory infected or uninfected insects is likely due to long-term selection and artificial rearing. Similarly, *T*. *rangeli* alters the midgut microbiome composition, and reduces the symbiont population levels in *R. prolixus* [246]. Some studies have reported that trypanosome infections did not affect the microbiota, but these studies assessed differences only in the hindgut or the whole-body [233,244].

The GI tract microbiota may limit the ability of trypanosomes to establish in the GI tract. The numbers of *T. cruzi* in aposymbiotic insects are ten times higher than in insects with a normal microbiota [247]. Some strains of *Serratia marcescens*, have direct trypanolytic activity [192,247,256,257], but this again only affects some *T*. *cruzi* strains as *S*. *marcescens* has been found in wild caught triatomines infected with *T*. *cruzi* [234,242,244,258]. Furthermore, in feral triatomines naturally infected with *T*. *cruzi,* there is an overrepresentation and subrepresentation of some *Actinomycetes* and Proteobacteria, but whether this helps or hinders *T*. *cruzi* infections remains to be addressed [234,241,245]. 

The triatomine immune system may play a role in these interactions. After blood feeding, and in insects fed with *T*. *cruzi*, *T*. *rangeli*, or bacteria, there is an activation of immune pathway genes, increases in AMP gene expression levels, increased levels of effector proteins and differential antimicrobial activity [246,247,249,255,259,260,261,262,263,264,265,266]. The AMPs involved in these studies are likely controlled by the Toll and IMD pathways as silencing the NF-κB transcription factor relish (IMD pathway) reduces the intestinal bacterial population while the opposite occurs when the NF-κB transcription factor dorsal (Toll pathway) is silenced [251]. The pharmacological inhibition of these pathways also affects AMP expression and reduces the microbiota levels [250]. Despite the effects on the microbiota, *T*. *cruzi* numbers remain unaltered when either pathway is silenced, suggesting that *T*. *cruzi* can tolerate the immune responses or actively induces some of these changes to regulate the microbiota [251,267]. 

## 5. Conclusions

Mutualistic symbiotic microorganisms are likely present in all insects, complementing nutritional deficiencies from the diet and providing fitness advantages to their hosts. There are diverse associations with primary symbionts in the Hemiptera. The Auchenorrhyncha, Sternorrhyncha, and Coleorrhyncha have predominantly ancient associations with intracellular primary symbionts while the Heteroptera (true bugs) from the Pentatomomorpha infraorder have established more flexible associations with extracellular intestinal bacteria. Triatomines have associations with extracellular intestinal beneficial symbionts, in contrast with other obligate hematophagous arthropods that rely on intracellular primary symbionts. Classical studies in triatomines have demonstrated the essential role of bacterial symbionts in the synthesis of specialized nutrients that are deficient in the hematophagous diet (such as vitamin B) or the supplementation of general nutritional resources. Microbiome studies of triatomines show a very diverse microbiota in the GI tracts. These demonstrate an overrepresentation of Nocardiaceae species, but no single bacterial species is present in all triatomines even within populations of the same species. This suggests that while intestinal bacteria are essential to triatomines, individual bacterial species are not, but some clades of bacteria have been selected commonly by triatomines. Multiple bacterial species may be selected as facultative secondary symbionts to establish a redundant backup system to ensure the health and wellbeing of the insect host. However, the mere presence of a bacterium in triatomines is not a conclusive indication of its role as a beneficial symbiont. Metabolomics studies will give deeper insight into these roles during critical stages in development. Intracellular bacteria such as *Wolbachia* sp. and *Arsenophonus* sp., are frequently found in triatomines but no parasitic or beneficial effects have been described. Overall, these studies have demonstrated complex and sometimes specialized interactions between triatomines and bacterial members of their microbiomes. Integrating the data from classical studies with novel approaches using microbiota identification, metabolomics, genomics, and bioinformatics will help us decipher the complex interactions and evolution of triatomine–microbial symbiont interactions.
